# High-Density Genetic Variation Map Reveals Key Candidate Loci and Genes Associated With Important Agronomic Traits in Peanut

**DOI:** 10.3389/fgene.2022.845602

**Published:** 2022-03-25

**Authors:** Huiling Zhao, Ruizheng Tian, Han Xia, Changsheng Li, Guanghui Li, Aiqin Li, Xianying Zhang, Ximeng Zhou, Jing Ma, Huailing Huang, Kun Zhang, Mahendar Thudi, Changle Ma, Xingjun Wang, Chuanzhi Zhao

**Affiliations:** ^1^ Institute of Crop Germplasm Resources (Institute of Biotechnology), Shandong Academy of Agricultural Sciences, Shandong Provincial Key Laboratory of Crop Genetic Improvement, Ecology and Physiology, Jinan, China; ^2^ College of Life Sciences, Shandong Normal University, Jinan, China; ^3^ College of Agricultural Science and Technology, Shandong Agriculture and Engineering University, Jinan, China; ^4^ Rajendra Prasad Central Agricultural University, Samsthipur, India

**Keywords:** peanut, GWAS, SNP array, molecular markers, agronomic traits

## Abstract

Peanut is one of the most important cash crops with high quality oil, high protein content, and many other nutritional elements, and grown globally. Cultivated peanut (*Arachis hypogaea* L.) is allotetraploid with a narrow genetic base, and its genetics and molecular mechanisms controlling the agronomic traits are poorly understood. Here, we report a comprehensive genome variation map based on the genotyping of a panel of 178 peanut cultivars using *Axiom_Arachis2* SNP array, including 163 representative varieties of different provinces in China, and 15 cultivars from 9 other countries. According to principal component analysis (PCA) and phylogenetic analysis, the peanut varieties were divided into 7 groups, notable genetic divergences between the different areas were shaped by environment and domestication. Using genome-wide association study (GWAS) analysis, we identified several marker-trait associations (MTAs) and candidate genes potentially involved in regulating several agronomic traits of peanut, including one MTA related with hundred seed weight, one MTA related with total number of branches, and 14 MTAs related with pod shape. This study outlines the genetic basis of these peanut cultivars and provides 13,125 polymorphic SNP markers for further distinguishing and utility of these elite cultivars. In addition, the candidate loci and genes provide valuable information for further fine mapping of QTLs and improving the quality and yield of peanut using a genomic-assisted breeding method.

## Introduction

Cultivated peanut (*Arachis hypogaea*. L) is one of the most important oil crops and cash crops in the world. In 2019, the total production of peanuts was approximately 48.8 million tons (http://www.fao.org). Peanuts are full of high-quality vegetable oil, protein, more than 30 essential vitamins, and many other nutrients, and is part of a balanced diet for human health. Peanuts are widely planted in India, China, United States, Argentina, Australia, and Brazil. In the past 20 years, the average yield of peanut increased from 1.95 t/ha in 1999 to 3.3 t/ha in 2019 in the above six countries (http://www.fao.org). However, peanut is often grown on marginal soils with lesser inputs and usually intercropped with cereals in many countries such as China and India, the top two countries with the largest peanut harvest area. At present, almost all peanut varieties are developed through conventional breeding methods, marker-assisted selection (MAS) technology was only used in a few traits in the peanut breed program, such as oleic acid content, and root-knot nematode resistance (Chu et al., 2011; Shasidhar et al., 2020). The average yield of peanut is significantly lower than that of the staple food crop, rice, and corn. Peanut germplasm resources have a narrow genetic background. It has been difficult to get significant improvement of the yield and quality through traditional cross-breeding. In the future, MAS will be an important alternative approach for increasing the yield and improving the quality of peanut.

Compared to the conventional breeding approach, MAS technology can significantly accelerate breeding process and improve breeding efficiency by increasing the genetic gains per selection cycles (Collard and Mackill, 2007; Varshney et al., 2013). The utility of MAS is becoming more and more popular in crop breeding programs. For example, in wheat, hundreds of resistance (R) genes to powdery mildew, leaf rust, and stripe rust have been mapped (Pinto da Silva et al., 2018; Qureshi et al., 2018; Shah et al., 2018), and many of them have been successfully used to improve the resistance of wheat through MAS. For MAS technology, identification of quantitative trait loci (QTL) or genes, and development of the closely linked markers is necessary. With the availability of genome sequence information and high-throughput genotyping technologies, genome-wide association study (GWAS) has become a powerful way to identify the tightly linked markers and QTLs from the genome, superseding the traditional QTL mapping method from the structured populations derived from two parents (Pujar et al., 2020). GWAS has been successfully used in identifying the QTLs and the key genes related with the complex traits on peanut. (Gangurde et al., 2020). In peanut, the markers associated with oil, protein, oleic acid, and linoleic acid through a preliminary GWAS analysis with 120 simple sequence repeat (SSR) and transposable element (TE) markers have been reported (Zhang et al., 2020b). Recently, using genotyping-by-sequencing based SNP markers, 79 loci significantly associated for the six yield-related traits were also reported (Zhou et al., 2021).

In the last decade, advances in high throughput sequencing and bioinformatics technologies provided a good platform for peanut genome research including marker development and trait mapping as well as development of molecular breeding products (Zhao et al., 2017; Han et al., 2018; Luo et al., 2018; Bertioli et al., 2019; Zhuang et al., 2019; Ma et al., 2020; Pandey et al., 2020; Zhao et al., 2020). The availability of large-scale genomic resources was used for identifying a large number of genome-wide SNPs, and high-throughput genotyping platforms like 48 K SNP array (*Axiom_Arachis*2, version II) (Clevenger et al., 2018) and 58K SNP array (Pandey et al., 2017). The 48 K SNP array is also used for GWAS analysis for 96 peanut genotypes and revealed that current Korean genetic resources lacked variability compared to US mini-core genotypes (Nabi et al., 2021). Zhang et al. reported the identification of 36 QTLs related with the 13 nutrient elements and 46 QTLs related with leaf spots resistance using the SNP array based GWAS analysis for 120 mini-core germplasms (Zhang H. et al., 2019; Zhang et al., 2020a). All these studies have successfully discovered loci associated with the agronomic traits of peanut.

In this study, we analyzed 20 phenotypic traits of 178 peanut cultivars from diverse origins in China and 9 other countries. We analyzed the genetic divergences of these peanut cultivars and identified a number of significant genetic loci related to phenotypic traits, which will be helpful for further fine mapping and genomic-assisted breeding.

## Materials and Methods

### Plant Materials

For genome-wide association study, a set of 178 peanut varieties (of which 119 varieties represented varieties from 13 provinces and regions including Shandong, Henan, Guangdong, and Fujian; 41 featured germplasm resources preserved in laboratories, and 18 were exotic, i.e., imported from resources abroad) were selected based on phenotypic characteristics, including plant height, number of total branches, seed and pod size, protein, etc. The botanical information for the selected Chinese varieties was derived from available monographs (Feng, 1987; Yu, 2008) and an online database (http://www.peanutdata.cn). The 178 peanut varieties consisted of *var. hypogaea*, *var. vulgaris*, *var. fastigiata*, *var. hirsuta,* and irregular type varieties. The detailed information of each sample was also listed on the Supplementary File S1.

### Phenotyping for Agronomic Traits

The test materials used were planted in the Jiyang Agricultural Planting base in Jinan City in the summer for 3 consecutive years from 2018 to 2020. The field experiment is a completely random design and adopts conventional cultivation management (Wan, 2003). At harvest, there are three peanut plants randomly selected from each peanut variety and the main stem height, lateral branch angle, total number of branches, pod length, seed length, linoleic acid content, and other traits were measured. The phenotypic data obtained were analyzed using Excel data analysis tools for descriptive statistics and normal distribution test, and Origin software was used for drawing.

### Genotyping of Peanut Cultivars

DNA was extracted from 15-day-old seedlings using Plant Genome Extraction Kit (Beijing, China), following the manufacturer’s instructions (https://www.tiangen.com/). The DNA was visualized in agarose gel containing Super GelRed (US Everbright Inc., Suzhou, China), and then quality and concentration were determined using Nanodrop™ 2000 spectrophotometer (Thermo Scientific, Shanghai, China). The second-generation 48K SNP array of peanut was used to obtain genotyping data of 178 materials (Clevenger et al., 2018). SNPs with low call rates were removed with selection criteria of missing data rate (>10%) and minor allele frequency (<5%). Only high-quality SNPs were selected for further analysis. Reference genome builds were acquired from arahy (https://peanutbase.org/peanut_genome).

### Population Genetic Analysis

The phylogenetic tree was constructed based on the SNPs identified above by maximum likelihood (ML) method in IQ-tree v1.6.12 (Minh et al., 2020) (http://www.iqtree.org/), which was visualized with ITOL software (Letunic and Bork, 2021) (https://itol.embl.de/). The bootstrap values were calculated with 1000 replicates. The population structure of the 178 samples was first evaluated using PCA by the GCTA package and later using Admixture v1.3.0 (Alexander et al., 2009). We used the default parameters in Admixture to test the number of ancestral populations (K) with a cross-validation (CV) process, and the one with minimum CV error calculated was selected as best K value (http://software.genetics.ucla.edu/admixture/admixture-manual.pdf) (Alexander et al., 2009), which was visualized in R script next.

### Genome-Wide Association Study

The TASSEL v5.2.1 software was used for the genome-wide association study (GWAS) analysis of 7 aforementioned yield and quality related traits with the high-quality SNPs (Bradbury et al., 2007). Both generalized linear model (GLM) and mixed linear model (MLM) were used to determine MTAs. In general, the GLM model focuses on the SNP effects, which only contains the fixed effects such as population structure and genotype, and the MLM model additionally adds random effects (kinship matrix) to correct for the cryptic relatedness. The Q-Q plots were used for selecting the best model of each trait. The Bonferroni-corrected *p*-value was used for mining the trait-related genome regions, and the markers that *p*-value of 0.05/13,125 (the total number of SNPs) or less were defined as significant. Based on the loci of MTAs, we used the online software - genome browser of peanut (https://www.peanutbase.org/gbrowse_peanut1.0) to screen the trait-related candidate genes among the trait-related regions.

## Results

### Phenotypic Analysis of Peanut Varieties

**FIGURE 1:**
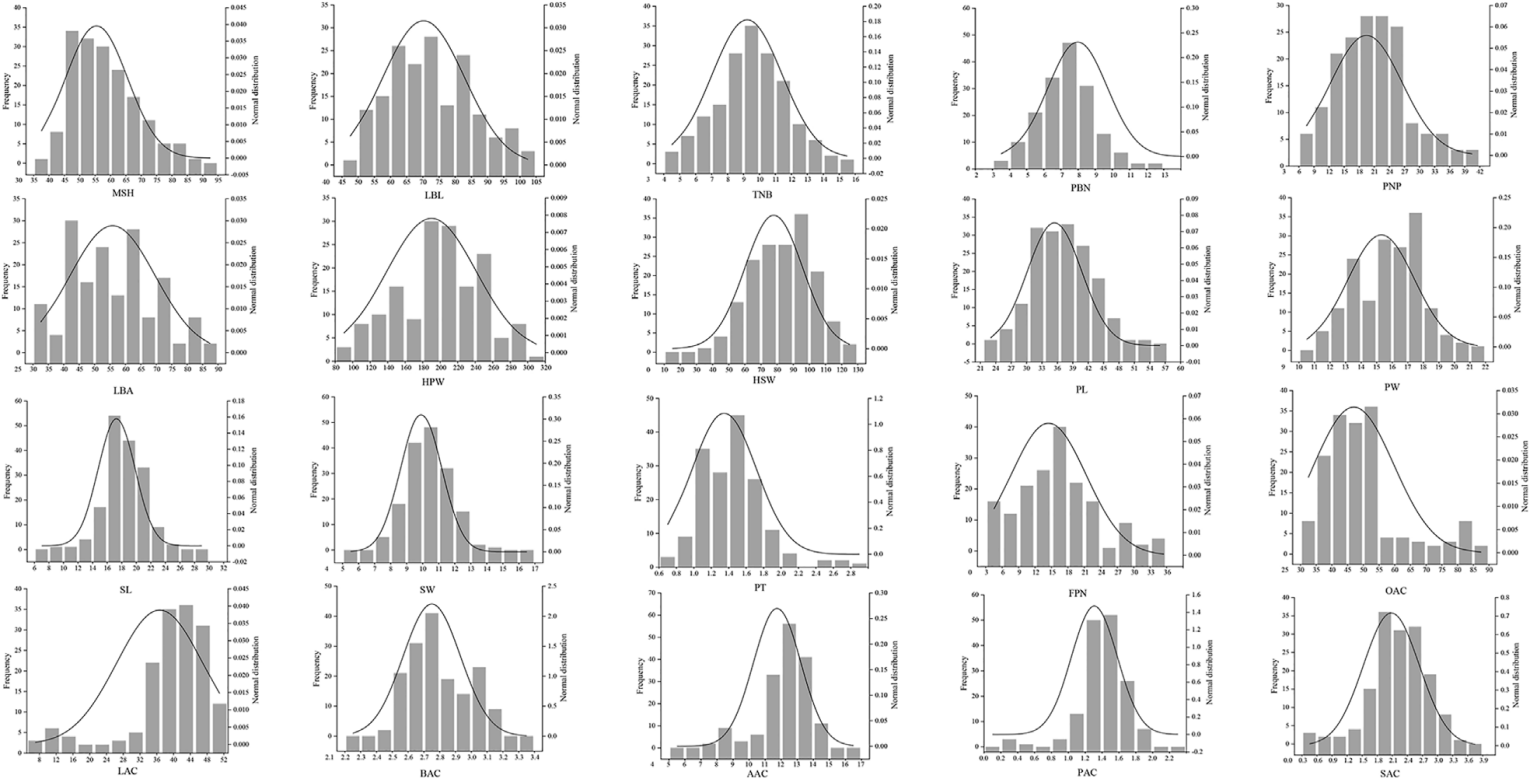
Frequency distribution of 178 peanut cultivars for 20 traits. MSH, main stem height; LBL, lateral branch length; TNB, total number of branches; PBN, pod-bearing branches number; PNP, pod number per plant; LBA, lateral branch angle; HPW, hundred pod weight; HSW, hundred seed weight; PL, pod length; PW, pod width; SL, seed length; SW, seed width; PT, peel thickness; FPN, filled pods number; OAC, oleic acid content; LAC, linoleic acid content; BAC, behenic acid content; AAC, arachidic acid content; PAC, palmitic acid content; SAC, stearic acid content.

Based on 3 years’ evaluation data, we observed a large phenotypic variability among 178 peanut cultivars for all the traits studied. Phenotype identification and statistical analysis showed that the 178 peanut genotypes displayed wide ranges of phenotypic variation for most of the agronomic traits. For example, the length of the lateral branch, the height of the main stem, and the angle of the lateral branch are important factors for determining the peanut plant type, which varied from 44.2 to 106.6 cm, 82.8–34.2 cm, and 30–90°, respectively (Table 1). Besides, many traits related to yield and quality as well as significant variation included the weight and the length of seed and the number of branches with the pod, and the latter varied from average 2.6 to 12 (Table 1). Moreover, the testa color also had high variation in these peanut varieties, and the seeds with pink, red, black, white, and variegation are included. In addition, many quality traits are also varied, including the content of oleic acid and linoleic acid (Table 1). Most of these traits accord with normal distributions indicating these traits could be quantitative traits (Figure 1).

**TABLE 1:** Phenotypic statistics of peanut major agronomic traits.

Traits	Abbreviation	Maximum	Minimum	Median	Mean	Standard deviation	Coefficient of variation (%)	Skewness	Kurtosis
Lateral branch length (cm)	LBL	99.0	44.20	66.00	67.73	12.72	19.08	0.40	−0.52
Main stem height (cm)	MSH	82.80	34.20	51.20	52.95	10.08	19.00	0.68	−0.01
Lateral branch angle (°)	LBA	90.00	30.00	50.00	53.32	13.82	25.91	0.33	−0.51
Total number of branches	TNB	16.40	3.60	8.80	8.72	2.19	25.16	0.29	0.59
Pod branching number	PBN	12.00	2.60	6.40	6.48	1.62	24.96	0.50	1.00
Pod number per plant	PNP	38.80	3.80	17.60	17.90	7.10	39.90	0.46	0.05
Hundred pod weight (gm)	HPW	329.20	57.20	194.40	190.05	51.04	26.86	−0.10	−0.20
Hundred seed weight (gm)	HSW	113.00	29.50	72.40	72.92	17.89	24.54	−0.11	−0.71
Pod length (mm)	PL	50.96	21.60	35.18	35.44	5.31	15.03	0.16	−0.29
Pod width (mm)	PW	20.02	10.01	14.97	14.79	2.05	13.80	−0.12	−0.51
Seed length (mm)	SL	23.23	7.63	17.16	17.30	6.36	14.10	−0.27	0.81
Seed width (mm)	SW	13.30	6.70	9.26	9.40	1.28	13.65	0.30	−0.18
Peel thickness	PT	2.69	0.52	1.25	1.25	0.37	30.70	0.99	2.07
Filled pods number	FPN	30.60	1.00	13.00	12.97	6.87	52.88	0.39	0.05
Oleic acid content (%)	OAC	82.02	28.51	42.15	42.15	12.36	27.78	1.41	1.63
Linoleic acid content (%)	LAC	49.15	4.66	38.81	36.69	10.27	27.99	−1.57	2.06
Behenic acid content (%)	BAC	3.08	2.37	2.66	2.64	0.18	6.70	0.33	−0.96
Palmitic acid content (%)	PAC	13.55	6.26	11.52	11.24	1.48	13.13	−1.40	1.97
Arachidic acid content (%)	AAC	1.75	0.12	1.23	1.21	0.27	22.47	−1.35	4.17
Stearic acid content (%)	SAC	3.07	0.05	2.00	1.93	0.56	28.39	−0.87	1.76

### Genome-Wide Distribution of SNP Markers

**FIGURE 2:**
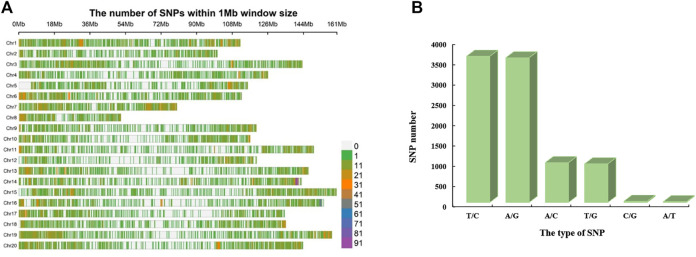
Distribution and types of SNPs. **(A)** Distribution and density of SNPs in 20 peanut chromosomes. The horizontal axis shows the length of the chromosome (Mb), and the vertical axis represents 20 chromosomes. The shades of assorted color represent the SNP density on corresponding loci. **(B)** Frequency of several types of SNPs.

The *Axiom_Arachis2* 48 K SNP array was used for genotyping the 178 peanut varieties (Nabi et al., 2021). A total of 34,712 SNPs were excluded based on filtering criterion: (1) SNPs with missing data rate (>10%) and (2) minor allele frequency (<5%). After filtration, 13,125 (27.43%) high quality SNPs were obtained (Supplementary File S2). On an average 4.69 SNPs/Mb were found distributed on 20 peanut chromosomes (Arahy.01 to Arahy.20) ranging from 3.74 SNPs/Mb to 7.84 SNPs/Mb (Table 2; Figure 2A). The maximum number of SNPs (800) were found on chromosome Arahy.14, followed by Arahy.01 (797) and Arahy.19 (788). As the smallest chromosome of the peanut genome, Arahy.08 contains only 340 SNPs. The density on chromosome Arahy.07 was the highest density (7.84 SNPs/Mb), while that on chromosome Arahy.12 was the lowest density (3.74 SNPs/Mb) (Table 2).

**TABLE 2:** Distribution and density of SNPs on 20 chromosomes and scaffolds of peanut.

Chromosome	Length of chromosome (Mb)	Number of SNPs	Density of SNPs
Arahy.01	112.42	797	7.09
Arahy.02	102.98	460	4.47
Arahy.03	143.81	684	4.76
Arahy.04	128.8	532	4.13
Arahy.05	115.93	503	4.34
Arahy.06	115.5	681	5.90
Arahy.07	81.12	637	7.84
Arahy.08	51.9	340	6.55
Arahy.09	120.52	546	4.53
Arahy.10	117.09	524	4.48
Arahy.11	149.3	569	3.81
Arahy.12	120.58	451	3.74
Arahy.13	146.73	628	4.28
Arahy.14	143.24	800	5.59
Arahy.15	160.88	758	4.71
Arahy.16	154.81	677	4.37
Arahy.17	134.92	572	4.24
Arahy.18	135.15	645	4.77
Arahy.19	158.63	788	4.97
Arahy.20	143.98	768	5.33
Scaffold		765	
Total	2538.29	13125	4.69

A total of six types of SNPs were observed including “T/C”, “A/G”, “A/C”, “T/G”, “C/G”, and “A/T”. We found that “T/C” is the most abundant type of SNPs, accounting for 39.43% of the total SNPs, followed by “A/G” which accounted for 39.05% of the total SNPs (Figure 2B). The “A/C” and “T/G” account for 10.71 and 10.37% of the total SNPs, respectively (Figure 2B). The “C/G” accounts for 0.29% of the total SNPs. The “A/G” is the least type of the SNPs, accounting for only 0.15% of the total SNPs (Figure 2B).

### Population Structure Analysis

**FIGURE 3:**
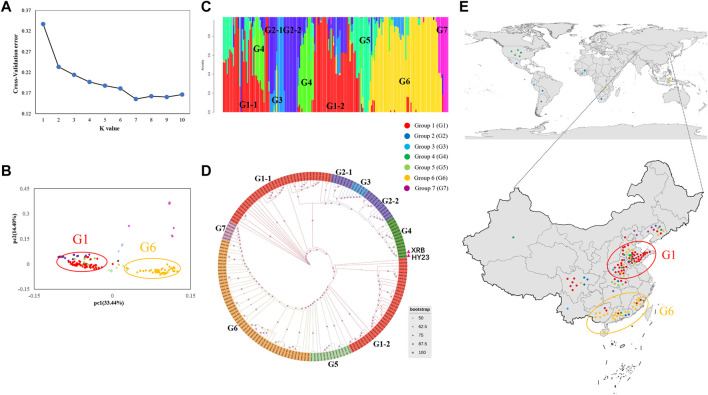
Population structure and genetic diversity of the 178 peanut varieties. **(A)** Cross-validation value of each K ranging from 1 to 10. **(B)** The PCA analysis of the total accessions. Each dot represents one variety. **(C)** Population structure. Each variety is represented with a single vertical line, and the color represents ancestry. **(D)** Phylogenetic trees constructed by the maximum likelihood method. **(E)** Geographical distribution of total varieties.

The population structure of the panel of peanut varieties was first investigated with the assessment of K value (Figure 3A), followed by validation via PCA (Figure 3B). The magnitude of CV error suggested that the best K (number of groups) was seven in the model-based group analysis. Based on their genotypes, the peanut panel could be divided into seven groups, group 1 (G1) to group 7 (G7), and the number of peanut varieties per group ranged from 5 to 72. The G1 containing 72 peanut varieties is the biggest group, followed by G6 which contains 53 peanut varieties (Figures 3C,D). Furthermore, the population structure and phylogenetic analysis results also suggested the presence of two subgroups of G1 (G1-1) and G2 (G2-1) (Figures 3C,D). The groups exhibited geographic distribution patterns, and the peanut varieties derived from the same planted areas of origin were usually in the same group. Most of the varieties originating from northern provinces including Shandong, Henan, and Hebei Provinces belong to G1, while the varieties originating from the southern provinces (Fujian, Guangdong, and Guangxi Provinces) were grouped into G6 (Figure 3E).

### Genome-Wide Association Study

Based on the Q-Q plot analysis, GLM was selected as the best model for GWAS signals among five traits: total number of branches, oil patch, peel thickness, main stem height and testa color; while the MLM was used for the other two traits: hundred seed weight (HSW) and pod shape (Supplementary Figure S1).

**FIGURE 4:**
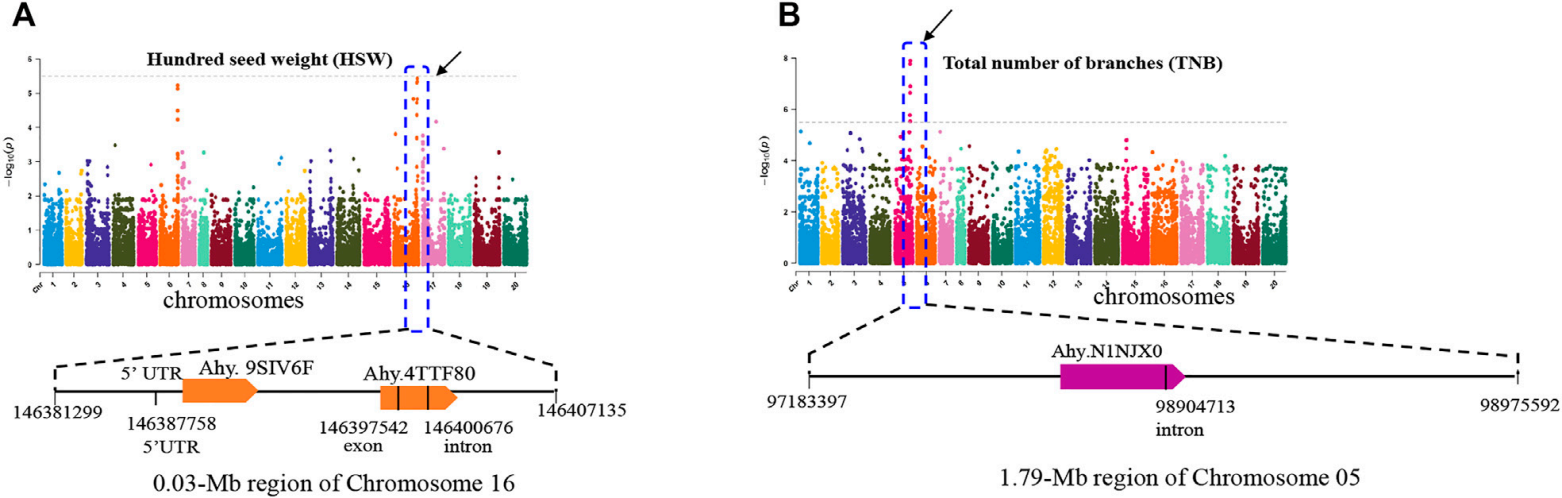
GWAS signals for hundred seed weight **(A)** and total number of branches **(B)** of peanut. The significance level is log10 (0.05/13125) = 5.4 (the gray horizontal line). The characteristic analysis of functional genes in the screening intervals is shown below each Manhattan plot.

Hundred seed weight and the total number of branches are important agronomic traits related to peanut yield. Under the threshold of -log10 > 5.4, MTA related to HSW was detected on chromosome 16 (Figure 4A). In addition, the associated SNPs were identified. The *SNP_Chr16:146387758* is located on the 5′UTR of the gene *Ahy. 9SIV6F* which encodes an unknown function protein. *SNP_Chr16:146400676* and *SNP_Chr16: 146397542* were all located in the gene region of *Ahy.4TTF80*, and the latter was in the exon of this gene. Function annotation showed that *Ahy.4TTF80* encodes an ABC-2 type transporter (Figure 4A). A previous study has shown that the ABC-2 type transporter protein was related with increasing size of plant seed and content of fat stored within the seed (Kim et al., 2013). For total number of branches, 1 MAT was detected in a 1.79-Mb region of chromosome 5 (97,904,713 to 98,975,592 bp), and 9 associated SNPs were enriched (Figure 4B). Among them, *SNP_Chr05:98904713* is in the intron of *Ahy.N1NJX0*, which is annotated as a calmodulin-binding transcription activator 2-like isoform X1. The other 8 SNPs were all located on intergenic regions (Figure 4B).

**FIGURE 5:**
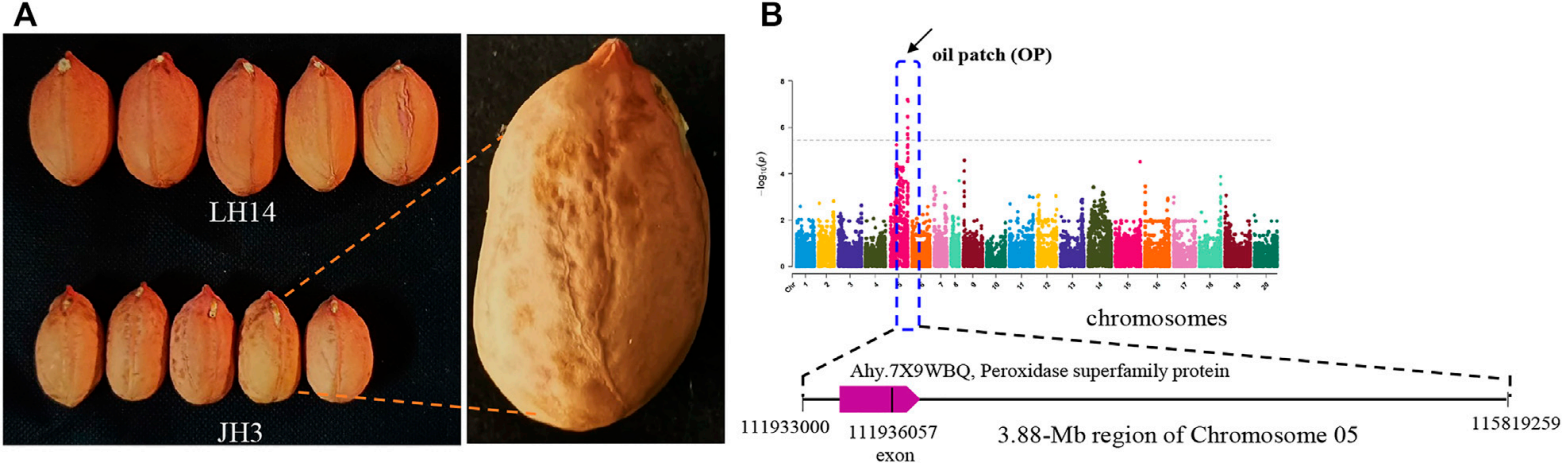
GWAS signals for oil patch (spots) of peanut. **(A)** Peanut cultivars without an oil patch (LH14) and with an oil patch (JH3). **(B)** Manhattan plot. The characteristic analysis for one gene encoding peroxidase superfamily protein is shown below.

**FIGURE 6:**
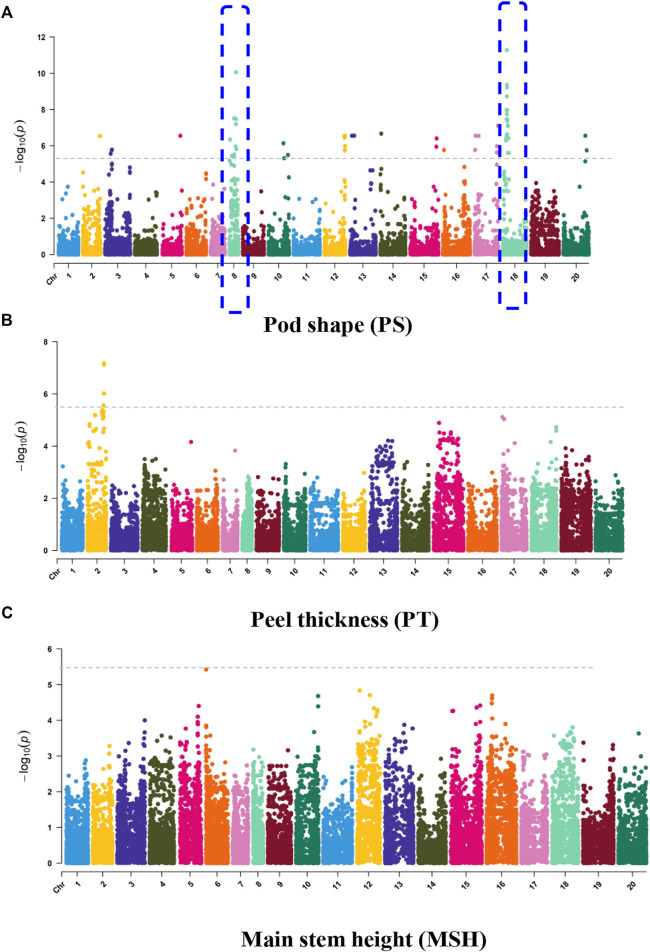
GWAS signals for **(A)** pod shape, **(B)** peel thickness, and **(C)** main stem height, related to the appearance of peanut.

The oil patch is the spot in the seed coat of the peanut (Figure 5A). The presence of an oil patch will affect peanut quality; however, the genetic and molecular mechanism of an oil patch are unclear. GWAS analysis showed that 6 SNPs were associated with the oil patch, and all of them were in a 3.88-Mb region of chromosome 5 ranging from chr.05:111.93–115.81 (Figure 5B). *SNP_Chr05:111936057* is on the exon of *Arahy.7X9WBQ*, which encodes a peroxidase superfamily protein. Pod shape, peel thickness, and testa color are also the important appearance traits of peanut. Pod shape is one of the important characteristics for the classification of peanut. A total of 14 MTAs were detected for pod shape, and distributed in Chr2, Chr3, Chr5, Chr8, Chr10, Chr12, Chr13, Chr14, Chr15, Chr16, Chr17, Chr18, and Chr20 (Figure 6A). Among them, the most significant association loci were detected on Chr8 and Chr18 (Figure 6A). For peel thickness, one MAT was detected on a 3.72 -Mb region of chromosome 2 (Chr2:86.18–89.45 Mb) (Figure 6B). For the main stem height, only one SNP was identified on chromosome 6 (Figure 6C).

**FIGURE 7:**
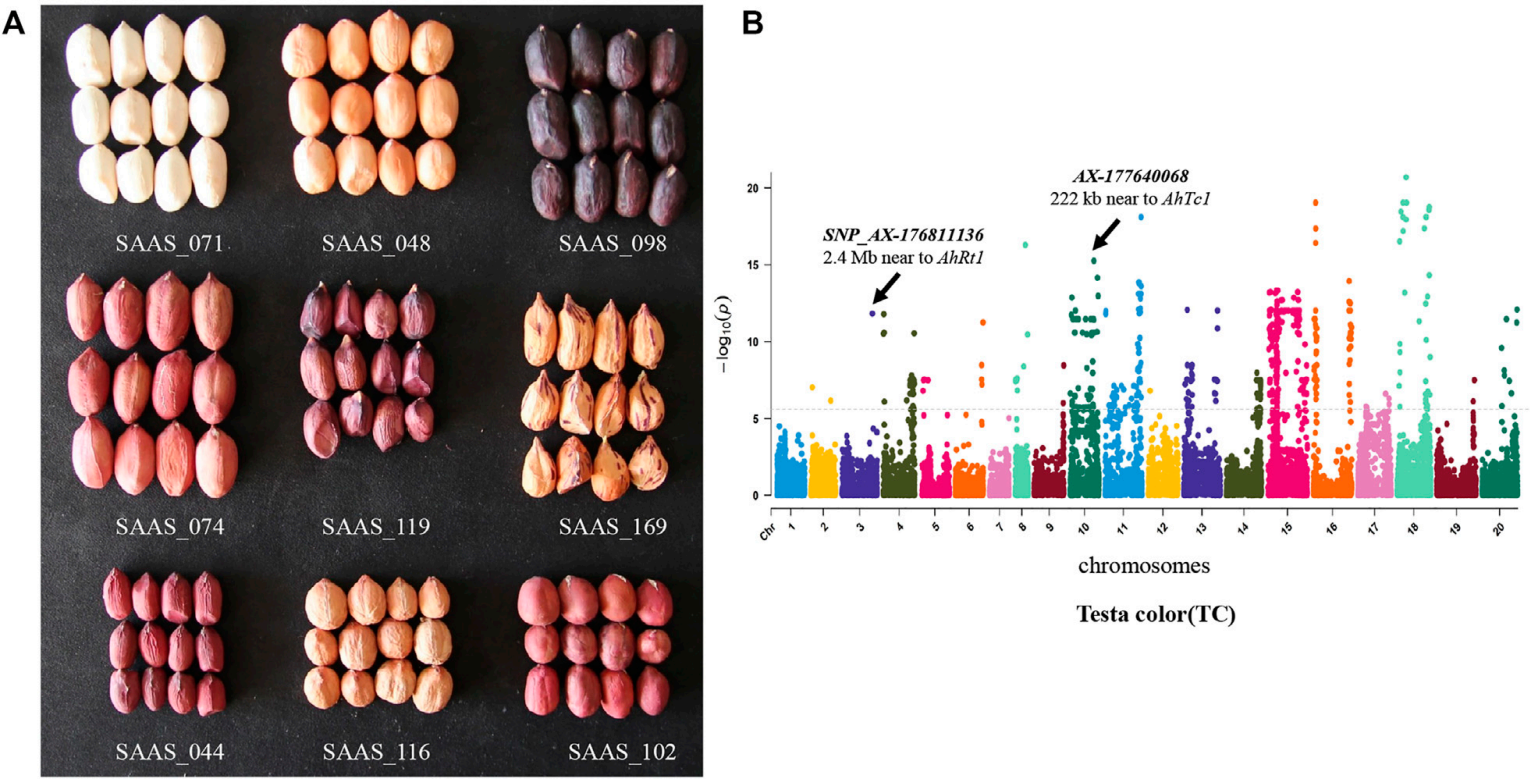
Manhattan plots showing significant marker-trait associations for testa color of peanut. **(A)** Peanuts with different testa color, and its **(B)** GWAS signal. The characteristic analysis for one gene encoding lysosomal cystine transporter is shown below.

The seed coat (testa) is an important trait of peanut which is not only as an important protective barrier for peanut seed against the pathogen, but also important for health nutrition such as anthocyanins and procyanidins. Testa color is also a complex trait which is controlled by at least 12 genes (Branch, 2011). In this study, the color of the 178 peanut genotypes displayed significant variations, including pink, red, black, purple, white, and variegation (Figure 7A). GWAS analysis showed that the associated SNPs were detected in most of the chromosomes except to Chr.01 and Chr.07 (Figure 7B). Among them, the *SNP_AX-177640068* of chr.10 is only 222 kb to the gene *AhTc1*, one of the key gene controlling black testa identified previously (Zhao et al., 2020). The *SNP_AX-176811136* in chr.03 is close to *AhRt1* locus contributing to red testa of peanut (Chen et al., 2021) (Figure 7B).

## Discussion

GWAS is a useful method for identifying linked loci and candidate genes by analyzing the association between the genotypes and the phenotypes of individuals in a population. Whole genome resequencing (WGS), high-density SNP array, and target genome resequencing (TGS) are the popular methods for acquiring the high throughput genotyping. WGS is with the maximum coverage across the genome, however, it is more expensive. TGS is a low-cost method that relies on the sequencing of target regions of the genome. Recently, several TGS methods have been developed and used in GWAS analysis, including genotyping by sequencing (GBS), restriction-site associated DNA sequencing (RAD-seq) and specific-locus amplified fragment sequencing (SLAF) (Zhang S. et al., 2019; Dodia et al., 2019; Wang et al., 2019; Yu et al., 2020; Jadhav et al., 2021). The SNP array is a low-cost and wide coverage method. The commercial high-density SNP array chips have developed in many crops, such as 660K of wheat (Hassan et al., 2021), 90K of rice (Yang et al., 2020), and 55K of maize (Li et al., 2021). In peanut, the available genome resource was used for identifying a large number of genome-wide SNPs, and large-scale 58 K SNP array (*Axiom_Arachis*) (Clevenger et al., 2017; Pandey et al., 2017) and 48K SNP array (*Axiom_Arachis2*) (Clevenger et al., 2018) have been developed. *Axiom_Arachis2* containing 47,837 SNPs is the second generation of peanut gene chip which has been successfully used for genetic diversity analysis and identification of QTLs related with the nutrient elements and leaf spots resistance of peanut (Zhang H. et al., 2019; Zhang et al., 2020a). However, the *Axiom_Arachis2* was developed prior to the release of a cultivated peanut genome. The positions of these SNPs were according to the genomes of wild type diploid peanut species, *A. duranensis* and *A. ipaensis*. In this study, we used *Axiom_Arachis2* to genotype 178 cultivars of peanut, and all of them are tetraploid cultivated peanut. Thus, we first remapped the probe sequences with the genome of cultivated peanut *Tifrunner* and updated the position information of the SNPs. In total, 45,608 SNPs were mapped in the 20 chromosomes of the peanut genomes and 2229 SNPs were mapped in the scaffolds. In the past few years, the SNP array has shown great potential for mapping the traits on peanut, and the updated position information of these SNPs will provide important references for future utility of the peanut SNP array (Pandey et al., 2021).

As an important index to evaluate seed size, HSW has been one of the hotspots in peanut genetics and QTL mapping. In this study, the HSW of the 178 peanut genotypes displayed wide ranges of variation, ranging from 29.5 to 113.0 g. Our results also showed that HSWs displayed variation within different groups. The G1 represented the varieties from Shandong, Henan, and Hebei Provinces. The average HSW of G1 is 85.1 g, which is significantly heavier than that of G6 (average HSW 66.0 g), in which most of the varieties come from southern provinces of China including Fujian, Guangdong, and Guangxi Provinces. Previous studies have reported many QTLs related with HSW, which is distributed in chr02, 03, 05, 07, 08, 12, 13, 14, 16, 17, and 18. Among them, QTLs in chr16 could be detected at least from four populations, including Fuchuandahuasheng × ICG 637, ZH16 × sd-H1, Zhonghua 16× J11, and Huayu 36 × 6–13, explaining up to 35.39% of the phenotypic variation (Huang et al., 2015; Wang et al., 2018; Mondal and Badigannavar, 2019; Zhang et al., 2019). In this study, the MTAs related with HSW were also detected in chromosome 16, and associated with the candidate gene *Ahy.4TTF80*, which encodes an ABC-2 type transporter. In tomato, the natural variation of the ABC transporter gene was associated with the seed size (Orsi and Tanksley, 2009). In rice, the ABC transporter gene, *OsABCG18* controls the shootward transport of cytokinin and is related with the grain yield of rice (Zhao et al., 2019). We found that there are two SNP substitutes in the *Ahy.4TTF80* gene, which provided an important clue for further fine mapped and revealed the key genes controlling the seed size of peanut. Besides, oil patch and pod shape are important agronomic traits of peanut. The oil patch and pod shape affect the appearance and commodity value. However, the physiology and genetics of them are rarely studied. In this study, we identified three MTAs related with the oil patch and pod shape. The details of those selected MTAs were shown in Supplementary Figure S3. These MTAs and candidate genes offer the opportunity to further study the molecular mechanism and improve these traits through the MAS approach.

Cultivated peanut is allotetraploid (AABB, 2n = 4 × = 40), derived from a hybridization event between *A. duranensis* (AA, 2n = 2 × = 20) and *A. ipaensis* (BB, 2n = 2 × = 20) about 3500 years ago (Kochert et al., 1996; Lavia et al., 2011). The molecular marker analysis demonstrated that cultivated peanut possesses a narrow genetic base (Halward et al., 1992), and some elite germplasm lines were overused in the peanut breeding program. For example, a previous study showed that more than 70% of peanut cultivars in China derived from two germplasms, Fuhuasheng and Shitouqi, directly or indirectly (Liao, 2004). In this study, a pedigree survey of these 120 peanut germplasms from 13 provinces and regions in China showed that 83 were from Fuhuasheng, accounting for 69.17%, and 42 were from Shitouqi, accounting for 35.00%. Thus, analysis of the genetic relationship between different germplasm resources is especially important for further designing hybrid combinations. In this study, we constructed the phylogenetic tree of these peanut germplasms through the 13,125 polymorphic SNPs. The results suggested that the geographical distribution is not exactly consistent with the genetic relationship among Chinese indigenous peanut breeds, which might be due to the exchange of germplasm resources across China (Figure 3). For example, Yueyou551 (SAAS_015) is classified into G1, however, it is a cultivar from the southern region of China. Pedigree analysis showed that the Yueyou551 derived from the cross-combination of Yueyou 22 and Yueyou 431, and the latter derived from the cross-combination of Shitouqi and Fuhuasheng. Fuhuasheng is a very typical elite peanut germplasm in the north of China. Besides, another two cultivars from the south, Tianfu3 (SAAS_128) and Guihua36 (SAAS_99), also have a close relationship with Fuhuasheng and is classified into G1. In addition, we found two peanut cultivars from Indonesia and one germplasm from Zambia (PI268586) are closely related to peanut varieties in south China, and classified into G6, which might be due to the exchange of germplasms between China and other countries. These results provide an important reference for further use of these germplasms.

## Conclusion

In this study, we analyzed 20 phenotypic traits of 178 peanut germplasms and genotyped them using the 48 K *Axiom_Arachis2* SNP array. We analyzed the genetic diversity of these cultivars and identified a number of MTAs related to different traits. The candidate SNPs and candidate genes for these MTAs are helpful for further fine mapping and improving the quality and yield of peanut via a molecular breeding method.

## Data Availability

The datasets presented in this study can be found in online repositories. The names of the repository/repositories and accession number(s) can be found below: NCBI's GEO database, accession number GSE197103.
