# Supplementary material for: High-Density Genetic Variation Map Reveals Key Candidate Loci and Genes Associated With Important Agronomic Traits in Peanut
Source: Front Genet. 2022 Mar 25;13:845602. doi: 10.3389/fgene.2022.845602 (PMC8990815; doi:10.3389/fgene.2022.845602)
Supplement: Supplementary file 3 [file Table1.DOCX]

Supplementary Material

**
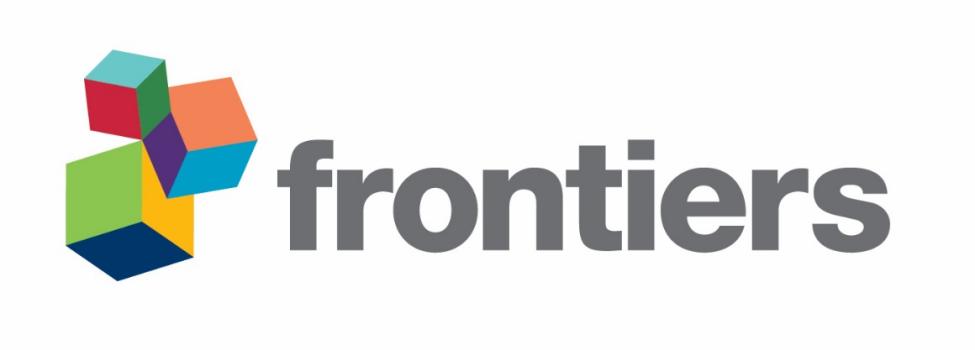
**

**Supplementary Figure 1.** Q-Q plots for GWAS signals. (A) hundred seed weight, (B) total number of branches, (C) oil patch, (D) pod shape, (E) peel thickness, (F) main stem height and (G) testa color.

**Supplementary File 1.** Detail information for the peanut varieties

**Supplementary File 2.** Polymorphic SNPs in 178 peanut materials

**Supplementary File 3.** Summary of the selected marker-trait associations (MTAs)
