# Supplementary figures and images for: High-Density Genetic Variation Map Reveals Key Candidate Loci and Genes Associated With Important Agronomic Traits in Peanut
Source: Front Genet. 2022 Mar 25;13:845602. doi: 10.3389/fgene.2022.845602 (PMC8990815; doi:10.3389/fgene.2022.845602)

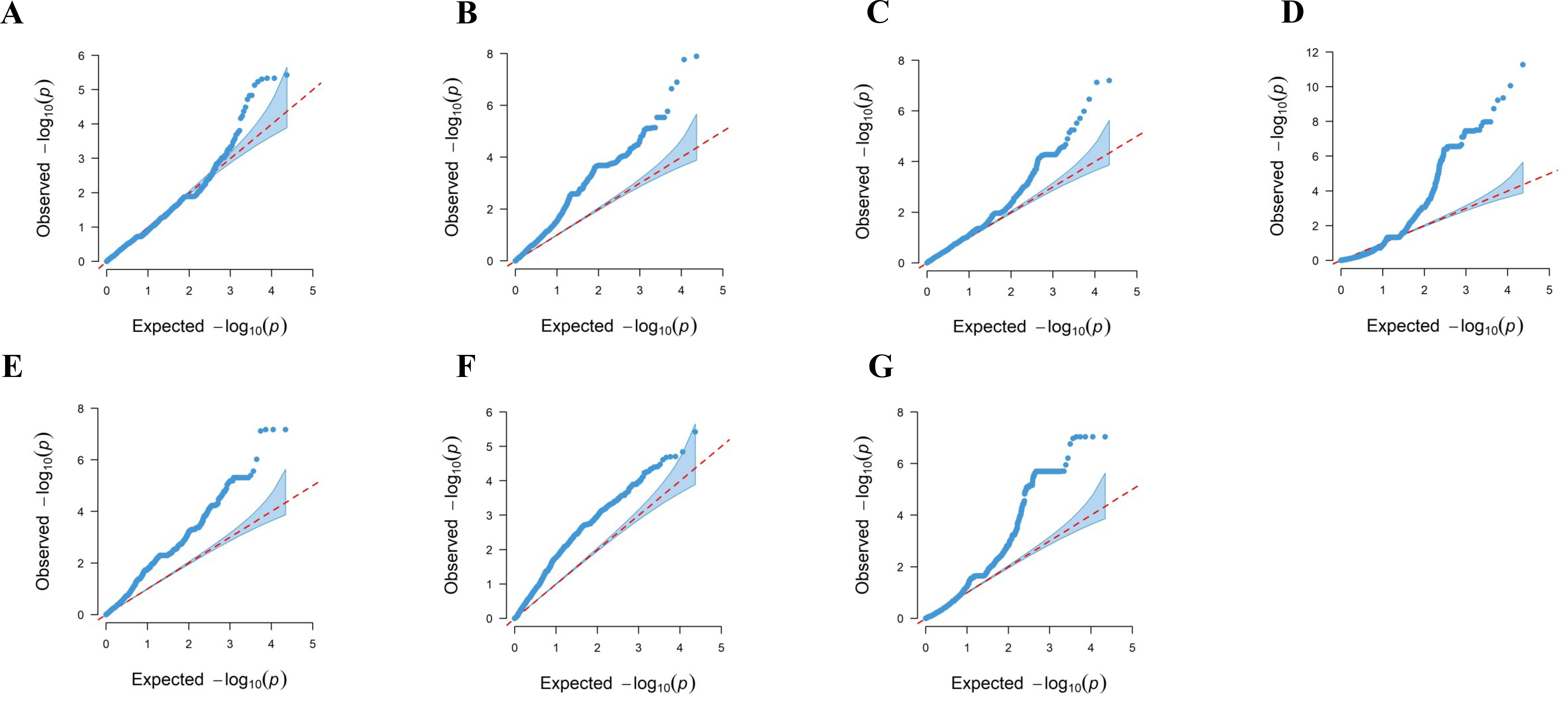

Supplement: Supplementary file 4 [file Image1.JPEG]
